# The identification of metabolites from gut microbiota in HPV infection via network pharmacology

**DOI:** 10.1371/journal.pone.0346716

**Published:** 2026-04-10

**Authors:** Wenbo Dong, Bai Li, Zhiwei Xu, Qi Wang, Zhihui Hou, Hongling Jia

**Affiliations:** 1 Second Affiliated Hospital of Shandong University of Traditional Chinese Medicine, Jinan, Shandong, China; 2 The Second Clinical Medical College, Shandong University of Traditional Chinese Medicine, Jinan, Shandong, China; 3 College of Acupuncture and Tuina, Shandong University of Traditional Chinese Medicine, Jinan, Shandong, China; Hong Kong Baptist University, HONG KONG

## Abstract

Human papillomavirus (HPV) infection continues to pose a significant global health challenge. Although gut microbial metabolites have been associated with HPV infection, the mechanisms underlying this relationship remain inadequately understood. A network pharmacology approach was utilized to comprehensively explore the connections between gut microbial metabolites and HPV infection. Using gutMGene, GeneCards, OMIM and other databases, 43 key targets were identified as common elements between gut microbial metabolites and HPV infection. Protein-protein interaction network analysis further screened 10 core targets, including IL6, AKT1, IL1B, CASP3, NFKB1, EGFR, PPARG, JUN, PTGS2, and TLR4. Gene ontology (GO) enrichment analysis of these 43 key targets indicated their involvement in lipopolysaccharide response, oxidative stress, and inflammatory signaling. Kyoto Encyclopedia of Genes and Genomes (KEGG) analysis of the 10 core targets highlighted the TNF, Toll-like receptor, C-type lectin receptor, and IL-17 signaling pathways as the main enriched pathways. A comprehensive microbiota-metabolite-target-pathway network was constructed, illustrating that these core targets interact with 13 gut microbial metabolites, 97 gut microbes, and 10 key pathways. Among the 13 metabolites screened, succinate and short-chain fatty acids (acetate, butyrate, propionate) exhibited favorable drug-likeness and toxicological profiles, with succinate being the most notable. Molecular docking analysis indicated that succinate binds to IL1B with a binding energy of −5.66 kcal/mol, suggesting a potential interaction with this core inflammatory target. These results suggest that key gut microbial metabolites, particularly succinate, may influence HPV infection through immune-related pathways and potential interactions with IL1B. This study provides a new direction for understanding the relationship between microbial metabolites and HPV infection and offers a foundation for future investigations.

## 1. Introduction

Human papillomavirus (HPV) infection is one of the most prevalent sexually transmitted infections globally and poses a substantial threat to public health [1]. HPV can infect human epidermis and mucous membranes, and serves as a major etiological factor for various epithelial tumors, such as cervical cancer, oropharyngeal cancer, and vulvar cancer. Nearly 90% of cervical cancers are directly attributed to persistent HPV infection [2,3]. Statistics show that there are more than 660,000 new cases of cervical cancer worldwide each year, with nearly 348,000 deaths, which poses a serious threat to women’s health [4]. HPV can achieve immune evasion by reshaping the host immune microenvironment, or influence the infection process by interfering with host metabolic pathways [5–7]. While prophylactic vaccines have reduced primary infections in young populations, existing interventions remain limited for already infected individuals, and there is a lack of effective biomarkers to monitor infection progression, especially those linking metabolic perturbations to disease dynamics [8].

In the field of HPV infection, existing studies have predominantly focused on changes in the vaginal microbiota [9]. The vaginal microbiota is undoubtedly the primary determinant of the local microenvironment in HPV infection. While the relationship between HPV and the vaginal microbiota was known to all, the vaginal microbiota primarily reflects the local cervical microenvironment. In contrast, the gut microbiota serves as the body’s largest immune reservoir and a central regulator of systemic metabolism. Its metabolites can enter the circulation and modulate immune responses at distant mucosal sites, including the cervix [10]. Therefore, exploring gut-derived metabolites may help elucidate the gut-cervix axis and its involvement in HPV infection.

However, recent studies have indicated that there is a correlation between the gut microbiota and HPV infection [11]. In recent years, the gut microbiota, referred to as the “second genome” of the human body, includes a diverse community of bacteria, fungi, and viruses, with Firmicutes and Bacteroidetes being the predominant phyla [12]. These microorganisms live in a cooperatively beneficial relationship with the host and play a crucial role in supporting the functional balance of the microbiome [13]. They are involved in processes such as host nutrient absorption, immune regulation, metabolism, and disease progression [14,15]. In a healthy state, they maintain a dynamic balance. Once this balance is disrupted beyond the body’s self-repair capacity, it can exert detrimental effects on the host, including chronic inflammation, autoimmune diseases, and even tumorigenesis [16]. A study found that Bacteroidetes is the main dominant bacterial taxon in the gut of patients with HPV-related cervical cancer [17]. Metabolites of the gut microbiome also play a role in HPV infection. Hydrogen sulfide, a metabolite produced by *Gemella*, is associated with intestinal barrier dysfunction and systemic inflammation. It can contribute to the persistent development of HPV infection by promoting immune dysregulation and viral persistence [18]. The gut microbiota can also regulate estrogen levels by secreting β-glucuronidase, which indirectly affects the relative abundance of *Lactobacillus* in the vagina, thereby influencing the progression of HPV infection [19]. Although the beneficial effects of gut microbial metabolites have been extensively studied, their pharmacological mechanisms on HPV have not been fully understood due to the complexity of metabolite-related targets and wide-ranging signaling pathways. Network pharmacology, a systems biology-based in silico approach, offers a new direction for investigating the interactions between targets, metabolites, and signaling pathways, thereby advancing our understanding of the mechanisms involved in HPV infection.

Using network pharmacology, this study investigated the relationships among gut microbiota, gut microbiome metabolites, targets, and HPV infection. The aim was to identify key gut microbiota, their metabolites, targets, and signaling pathways involved in the progression of HPV infection, and to explore gut microbiome metabolites with potential relevance to HPV infection. Based on the limited availability of genotype-specific data in public databases, we focused on HPV infection broadly without stratification by genotype, aiming to initially identify core targets and pathways that may be commonly involved in HPV pathogenesis.

## 2. Methods

### 2.1. Study design

**Fig 1:**
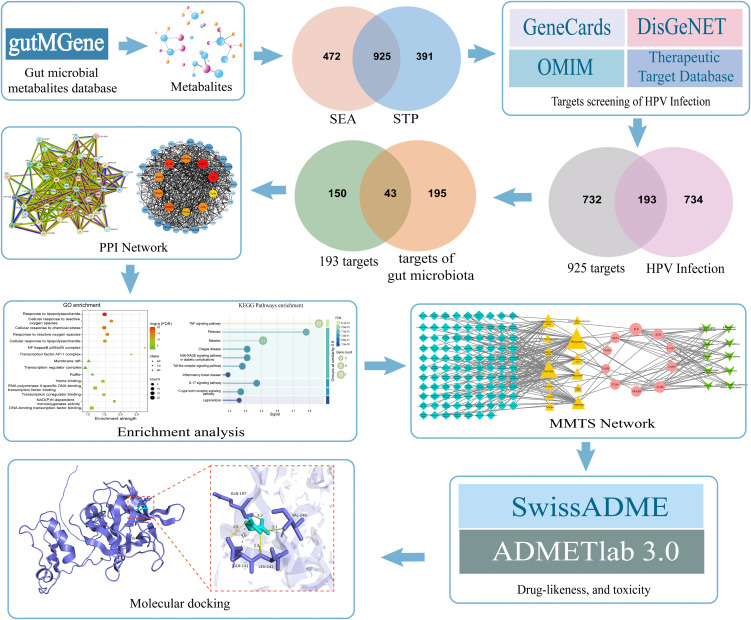
Flow chart of the study. Study workflow integrating multi-database screening, network analysis, enrichment analysis, and molecular docking to identify HPV-related gut microbial metabolites.

Using network pharmacology, this study explored the potential regulatory mechanisms of gut microbial metabolites on the progression of human papillomavirus (HPV) infection. A complex regulatory network between gut microbiota and their metabolites, and HPV infection was constructed, and gut microbial metabolites with therapeutic potential were screened. This work provides a new research direction for the development of intervention strategies against HPV infection (Fig 1).

### 2.2. Selection of gut microbiota metabolites and HPV infection targets

Gut microbiota, their metabolites, and targets information was extracted from the gutMGene V2.0 database (http://bio-computing.hrbmu.edu.cn/gutmgene; accessed on 21 June 2025). GutMGene V2.0 provides strain-level resolution and experimental traceability, with associations curated from published studies or derived from human gut microbial reference genomes. The database records information, including rank, host species, sample type, experimental method, measurement technique, and PubMed ID [20]. From this database, we obtained information on the human gut microbiota, their metabolites, and associated targets. The SMILES (simplified molecular input line entry system) of these metabolites were retrieved via PubChem (https://pubchem.ncbi.nlm.nih.gov/; accessed on 22 June 2025), then input into the Similarity Ensemble Approach (SEA) database [21] (http://sea.bkslab.org/; accessed on 24 June 2025) and SwissTargetPrediction (STP) database [22] (http://swisstargetprediction.ch/; accessed on 24 June 2025) with the species set to “*Homo sapiens*”, to predict metabolite-related targets. Using the Venny tool on the Wei Sheng Xin platform (https://bioinfogp.cnb.csic.es/tools/venny/index.html; accessed on 24 June 2025), a VENN diagram was generated to intersect the results from SEA and STP databases, yielding the targets associated with gut microbial metabolites.

Using “HPV infection” as the keyword, we retrieved targets associated with HPV infection from the GeneCards database [23] (https://www.genecards.org/; accessed on 24 June 2025), OMIM database [24] (https://www.omim.org/; accessed on 24 June 2025), Therapeutic Target Database [25] (https://db.idrblab.net/ttd/; accessed on 24 June 2025), and DisGeNET database [26] (https://disgenet.com/; accessed on 24 June 2025). For GeneCards, targets with a “relevance score” above the median were selected. After integrating these four databases and removing duplicate entries, a HPV infection target database was constructed. Standard target names were obtained using the UniProt Knowledgebase (https://www.uniprot.org/; accessed on 24 June 2025), and the species was selected as “*Homo sapiens*”. A VENN diagram was employed to intersect microbial metabolite-related targets with HPV infection targets, identifying important targets of microbial metabolites that act on HPV infection. Finally, these important targets were further intersected with those in the gutMGene database to obtain key targets.

### 2.3. PPI network construction and enrichment analyses

Key targets were uploaded to the STRING database [27] (https://cn.string-db.org/; accessed on 25 June 2025) to construct a protein-protein interaction (PPI) network, with relevant data downloaded for subsequent analyses. The network was visualized using Cytoscape 3.7.2 [28]. To further identify core targets through which gut microbial metabolites influence HPV infection, the CytoHubba plugin [29] in Cytoscape 3.7.2 was used to screen core genes, with the 10 core targets exhibiting the highest scores selected via the degree algorithm.

Gene Ontology (GO) enrichment analysis was performed on the 43 key targets using the STRING database to characterize biological functions relevant to gut microbial metabolites and HPV progression, including biological processes (BP), cellular components (CC), and molecular functions (MF). Significantly enriched terms were identified using a threshold of false discovery rate (FDR) < 0.05, calculated by the Benjamini-Hochberg procedure. Significantly enriched terms within each GO category were ranked by the signal score provided by the STRING database. The top 5 terms from each category were selected for bubble plot visualization. Additionally, the 10 core targets from the PPI network were analyzed via KEGG pathway enrichment using the STRING database, with the same FDR < 0.05 threshold. The significantly enriched pathways were then ranked by the signal score. The top 10 significantly enriched pathways were selected for visualization.

### 2.4. Construction of the microbiota-metabolite-target-signaling pathway (MMTS) network

An MMTS network was constructed using Cytoscape 3.7.2 software to visualize gut microbiota, metabolites, targets, and signaling pathways. This network facilitates understanding of the functions and interaction mechanisms of each component within the biological system underlying HPV infection.

### 2.5. Druglikeness evaluation

The physicochemical properties of relevant metabolites were simulated using the SwissADME platform [30] (http://www.swissadme.ch/; accessed on 1 July 2025) to assess their druglikeness [22]. Candidate metabolites involved in HPV infection progression were prioritized based on Lipinski’s rules: molecular weight (MW) ≤ 500 Da, hydrogen bond acceptors (HBA) ≤ 10, hydrogen bond donors (HBD) ≤ 5, molecular lipophilicity potential (MLogP) ≤ 5, and topological polar surface area (TPSA) < 140 Å².

### 2.6. Toxicological evaluation via ADMETlab

Candidate metabolites were subjected to toxicological profiling using the ADMETlab 3.0 platform [31] (https://admetlab3.scbdd.com/; accessed on 1 July 2025) across eight parameters: hERG channel blockers, drug-induced liver injury (DILI), human hepatotoxicity (H-HT), rat oral acute toxicity, skin sensitization, carcinogenicity, genotoxicity, and Ames test. These analyses facilitated the identification of gut microbial metabolites involved in HPV infection progression with the most significant research value.

### 2.7. Molecular docking validation of gut microbiota metabolites

To validate potential interactions, molecular docking was used between gut microbial metabolites and their core targets. The metabolite structures were prepared using the Yinfo Cloud Computing Platform (https://cloud.yinfotek.com/; accessed 31 January 2026). To avoid issues with missing residues common in crystallographic data, core target structures were obtained from the AlphaFold Protein Structure Database [32] (https://alphafold.com/; accessed 31 January 2026). Molecular docking was performed with AutoDock 1.5.6 using a grid box of 112 × 84 × 126 Å centered at x = −2.471, y = 4.408, and z = −16.418, covering the active pocket. Conformational sampling used the Lamarckian Genetic Algorithm, and optimal poses were selected based on binding energy and interaction type. Complexes were visualized with PyMOL.

For protocol validation, crystal structures from the Protein Data Bank (PDB; https://www.rcsb.org/; accessed 31 January 2026) were used. Co-crystallized ligands were redocked into their original pockets, and the RMSD between predicted and experimental poses was calculated. An RMSD below 2.0 Å confirmed the reliability of the docking parameters. Positive inhibitors were also docked as controls under the same conditions using AlphaFold-predicted target structures, providing reference binding energies for comparison [33].

## 3. Results

### 3.1. Targets of gut microbiota metabolites acting on HPV infection

**Fig 2:**
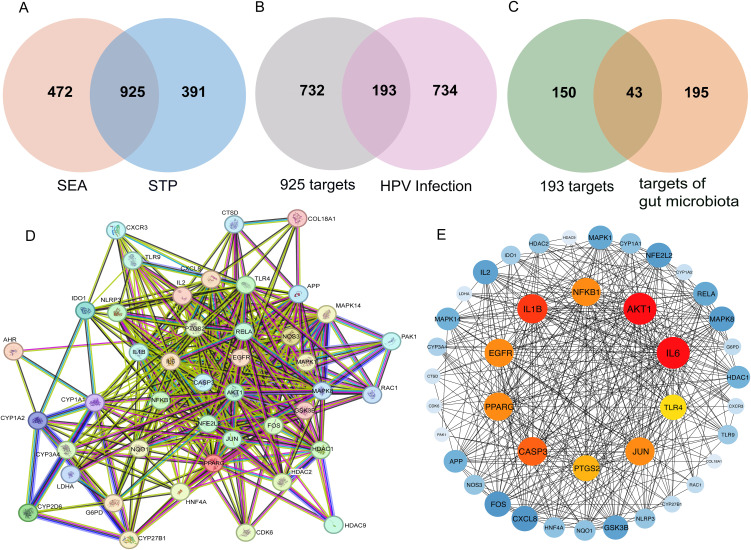
Identification of potential targets shared between gut microbiota metabolites and HPV infection. (A) 925 overlapping targets of gut microbial metabolites between SEA and STP databases; (B) 193 common targets between 925 metabolite targets and HPV infection-related targets; (C) 43 common targets between 193 gut microbiota metabolism-HPV infection targets and gut microbiota targets; (D) PPI network from STRING. The network has 43 nodes and 412 edges; (E) Ten core targets of the PPI network. The CytoHubba plugin was used to identify core targets based on the Degree algorithm. The top 10 genes with the highest Degree values were selected as core targets and highlighted in red. In the network visualization, node size and color intensity are proportional to the Degree value, with larger and darker nodes representing higher Degree scores.

The gutMGene V2.0 database encompasses 282 human gut microbial species, 278 gut microbiota-derived metabolites, and 238 host targets. These retrieved metabolites were uploaded as SMILES strings to the SEA and STP databases, resulting in 1,397 and 1,316 predicted targets, respectively. Intersection analysis of these datasets identified 925 overlapping targets (Fig 2A).

Targets were compiled and deduplicated from the GeneCards Database, OMIM Database, Therapeutic Target Database, and DisGeNET Database, resulting in 927 targets. These targets were cross-referenced with the 925 targets of the aforementioned metabolites, yielding 193 significant targets (Fig 2B). Finally, these 193 significant targets were further intersected with 238 host targets from the gutMGene database, ultimately identifying 43 key targets of gut microbial metabolites involved in persistent HPV infection (Fig 2C).

### 3.2. Construction of PPI network

The key targets were uploaded to the STRING platform for PPI analysis, and the resulting PPI network identified a total of 43 nodes and 412 edges (Fig 2D). To further determine the core targets through which gut microbial metabolites regulate HPV infection, we utilized the cytoHubba plugin in Cytoscape 3.7.2 to screen the top 10 core targets based on degree values. The results indicated that IL6, AKT1, IL1B, CASP3, NFKB1, EGFR, PPARG, JUN, PTGS2, and TLR4 are the 10 core targets in the PPI network (Fig 2E).

### 3.3. GO enrichment analysis

**Fig 3:**
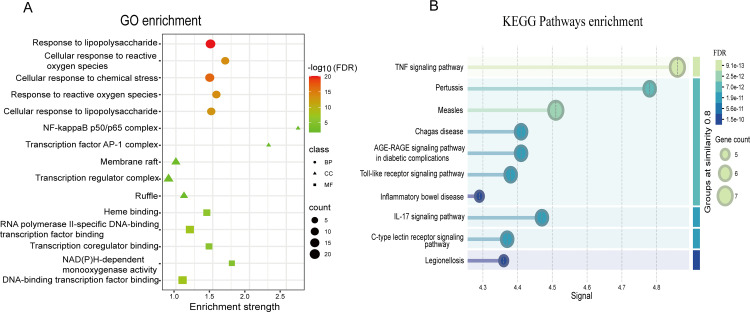
GO and KEGG enrichment analyses of gut microbiota metabolite-related targets. (A) GO enrichment analysis of 43 key targets. Bubble size represents gene count. Color gradient reflects -log10(FDR), with darker colors indicating higher statistical significance. (B) KEGG pathway enrichment analysis of 10 core targets. Bubble size indicates gene count. Color represents FDR significance, with lighter colors (mint) corresponding to lower FDR values (more significant enrichment). Pathways are ranked by enrichment signal score.

To further evaluate the potential mechanisms of gut microbiome metabolites in therapeutic strategies for HPV infection, the enrichment analysis on the 43 key targets was performed. GO enrichment analysis of these 43 key targets was conducted using the STRING database, with a screening criterion of FDR < 0.05 (S1 Table in S1 Data). The GO analysis results revealed that the significantly enriched Biological Process terms were predominantly associated with cellular stress responses and immune recognition, including response to lipopolysaccharide, cellular response to lipopolysaccharide, cellular response to reactive oxygen species, cellular response to chemical stress, and response to oxidative stress. The cellular components involve NF-kappaB p50/p65 complex, transcription factor AP-1 complex, membrane raft, transcription regulator complex, and ruffle. The molecular functions cover activities such as heme binding, RNA polymerase II-specific DNA-binding transcription factor binding, transcription coregulator binding, NAD(P)H-dependent monooxygenase activity, and DNA-binding transcription factor binding (Fig 3A).

### 3.4. KEGG enrichment analysis

Based on the biological processes involved in the aforementioned core targets, gut microbial metabolites may exhibit potential therapeutic effects by regulating HPV infection-related pathological processes, such as inflammatory cascades and host immune responses. To further dissect the underlying molecular pathways, KEGG signaling pathway enrichment analysis was performed on the core targets. Using the signal value reflecting the signal intensity of pathway enrichment as the screening criterion, the top 10 major pathways were identified (S2 Table in S1 Data), including the TNF signaling pathway, Toll-like receptor signaling pathway, C-type lectin receptor signaling pathway, and IL-17 signaling pathway (Fig 3B). The results of KEGG enrichment analysis can be categorized into the following aspects: signal transduction (TNF signaling pathway, Toll-like receptor signaling pathway, C-type lectin receptor signaling pathway, IL-17 signaling pathway, AGE-RAGE signaling pathway in diabetic complications), human diseases (pathways related to Measles, Pertussis, Chagas disease, Legionellosis, and Inflammatory bowel disease), and biological systems (immune system and endocrine system).

### 3.5. Construction of MMTS network and screening of key components

**Fig 4:**
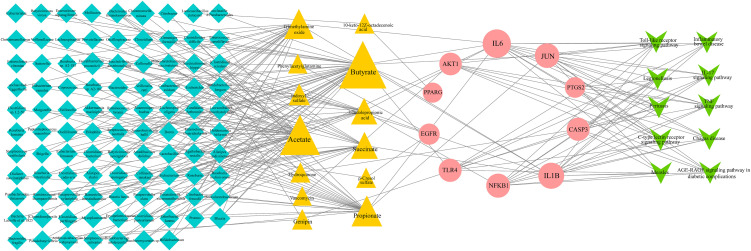
MMTS network. The network comprises 130 nodes and 225 edges, illustrating the complex relationships between gut microbiota, their metabolites, core targets, and signaling pathways. Blue diamonds represented gut microbiota; Pink triangles represented gut microbiome metabolites; Yellow circle represented targets; Green V-shaped represented Signaling pathways. Node size is proportional to the degree score, with larger nodes indicating greater connectivity within the network.

MMTS network analysis was performed using Cytoscape 3.7.2 software, which identified a total of 130 nodes (97 gut microbiota, 13 gut microbial metabolites, 10 core targets, and 10 pathways) and 225 edges (Fig 4). Network analysis revealed that the most critical signaling pathways include: the Toll-like receptor signaling pathway, which connects with 6 targets (IL1B, NFKB1, JUN, TLR4, IL6, AKT1) and 7 metabolites; the IL-17 signaling pathway, linked to 6 targets (IL1B, NFKB1, CASP3, PTGS2, JUN, IL6) and 8 metabolites; the C-type lectin receptor signaling pathway, associated with 6 targets (NFKB1, IL1B, PTGS2, JUN, IL6, AKT1) and 7 metabolites; and the TNF signaling pathway, connected to 7 targets (IL1B, NFKB1, CASP3, PTGS2, JUN, IL6, AKT1) and 9 metabolites (Table 1).

**Table 1:** Gut microbiota metabolites and core targets of major signaling pathways.

Signaling pathways	Targets	Metabolites
hsa04620: Toll-like receptor signaling pathway	NFKB1, IL1B, JUN, TLR4, IL6, AKT1	Succinate, Butyrate, 3-Indolepropionic acid, Vancomycin, Trimethylamine oxide, Acetate, Propionate
hsa04657: IL-17 signaling pathway	NFKB1, IL1B, CASP3, PTGS2, JUN, IL6	Succinate, Butyrate, 3-Indolepropionic acid, Trimethylamine oxide, Acetate, Genipin, Propionate, Hydroquinone
hsa04625: C-type lectin receptor signaling pathway	NFKB1, IL1B, PTGS2, JUN, IL6, AKT1	Succinate, Butyrate, 3-Indolepropionic acid, Vancomycin, Trimethylamine oxide, Acetate, Propionate
hsa04668: TNF signaling pathway	NFKB1, IL1B, CASP3, PTGS2, JUN, IL6, AKT1	Succinate, Butyrate, 3-Indolepropionic acid, Trimethylamine oxide, Acetate, Genipin, Propionate, Hydroquinone, Vancomycin

### 3.6. In silico identification of drug-likeness

The ADME parameters of 13 metabolites were analyzed. However, due to the excessive length of the SMILES format for Vancomycin, relevant physicochemical property results could not be obtained from the SwissADME platform. Therefore, a literature review was conducted, which indicated that this metabolite possesses drug-like properties. Additionally, analysis showed that the remaining 12 metabolites all conform to Lipinski’s rule, indicating favorable drug-like physicochemical properties. This suggests that these metabolites may possess the potential to influence HPV infection (Table 2).

**Table 2:** The physicochemical properties of the metabolites from gut microbiota.

Metabolites	Lipinski rules				Lipinski’s violations ≤1	Bioavailability score >0.1	TPSA <140 Å^2^
	MW ≤ 500	HBA ≤ 10	HBD ≤ 5	MlogP ≤ 5			
Acetate	59.04	2	0	−0.49	0	0.85	40.13
Trimethylamine oxide	75.11	1	0	−1.66	0	0.55	29.43
Succinate	116.07	4	0	−0.54	0	0.56	80.26
Genipin	226.23	5	2	0.12	0	0.56	75.99
Hydroquinone	110.11	2	2	0.79	0	0.55	40.46
P-Cresol sulfate	188.2	4	1	1	0	0.85	71.98
10-keto-12Z- octadecenoic acid	296.44	3	1	3.59	0	0.85	54.37
Butyrate	87.1	2	0	0.49	0	0.85	40.13
Propionate	73.07	2	0	0.03	0	0.85	40.13
Indoxyl sulfate	213.21	4	2	0.22	0	0.56	87.77
3-Indolepropionic acid	189.21	2	2	1.4	0	0.85	53.09
Phenylacetylglutamine	264.28	4	3	0.4	0	0.56	109.49

Note: All data were predicted using the SwissADME platform, except for vancomycin, whose properties were obtained from literature review [34,35].

### 3.7. Toxicological properties of 13 metabolites

To ensure the safety of metabolites, evaluation of their toxicological properties is crucial. Therefore, building on the identification of drug-likeness, toxicological assessment of the selected 13 metabolites was performed using ADMETlab 3.0 (S3 Table in S1 Data). Finally, succinate was identified as the most promising metabolite, which may hold significant research and development value.

### 3.8. Molecular docking validation

In molecular docking, binding free energy serves as a fundamental metric for quantifying intermolecular affinities. A docking binding energy less than 0 kcal/mol indicates that the receptor and ligand can bind spontaneously, suggesting a favorable binding potential. Notably, a binding affinity below −5.0 kcal/mol is generally regarded as a threshold for robust binding potential. A lower binding energy indicates superior conformational stability and higher occupancy of the binding site, which collectively enhance the likelihood of triggering downstream biochemical cascades.

**Fig 5:**
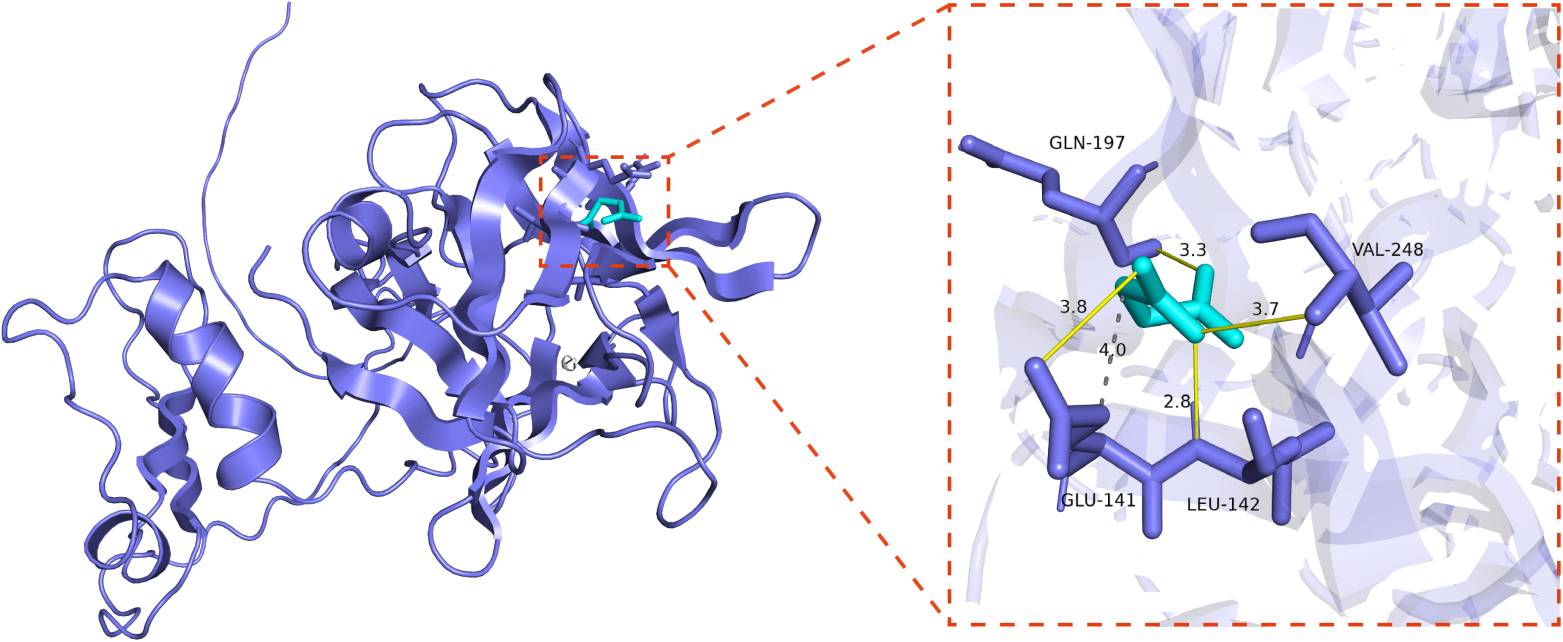
Molecular docking assays for the core target IL1B-succinate pair. Succinate binds to the active site of IL1B, forming hydrogen bonds with GLN-197, VAL-248, GLU-141, and LEU-142.

In silico analysis revealed that succinate exhibits robust binding affinity for IL-1B, yielding a docking score of −5.66 kcal/mol (Fig 5), which marginally outperformed the established positive control, quercetin (−5.55 kcal/mol; S1 Fig in S1 Data.). This suggests a favorable interaction between the metabolite and the target protein. To ensure methodological precision, a re-docking validation was performed using the IL-1B crystal structure (PDB: 5R87). The predicted binding pose of the native ligand showed excellent congruence with its experimental orientation, with a RMSD of 0.509 Å. This value, well below the standard 2.0 Å threshold, confirms the fidelity of the force field and the predictive accuracy of our docking parameters.

## 4. Discussion

HPVs represent one of the most genetically diverse families of viruses infecting humans. Fortunately, among the numerous HPV subtypes, only a small subset of high-risk types possess oncogenic potential. Persistent infection with these high-risk types can lead to epithelial malignancies, including cervical and oropharyngeal cancers, which remain leading causes of cancer-related mortality among women globally [36]. Current clinical interventions for HPV infection remain notably limited: for susceptible populations, invasive treatments such as loop electrosurgical excision, conization, and cryotherapy are commonly employed, or reliance is placed on interferon-α, therapeutic vaccination, with few oral medication options [37]. As “invisible regulators” of host health, gut microbiota and their metabolites may serve as potential intervention targets by modulating the pathogenesis of HPV infection. Therefore, network pharmacology was employed to comprehensively elucidate the complex interactions of gut microbial metabolites in regulating the MMTS network during HPV infection, aiming to identify potential biomarkers for intervening in the progression of HPV infection. Our findings suggest that succinate may represent a metabolite of interest warranting further experimental investigation.

The PPI network identified ten core targets (IL6, AKT1, IL1B, CASP3, NFKB1, EGFR, PPARG, JUN, PTGS2, and TLR4), which are deeply involved in the immune dysregulation, sustained inflammation, and aberrant cell proliferation that characterize HPV infection progression. Among these ten targets, IL6, IL1B, PTGS2, and NFKB1 act as central nodes in pro-inflammatory signaling. IL6, a pleiotropic cytokine, is elevated in HPV-infected cervical tissues and fuels inflammation and malignant transformation, partly via STAT3 activation [38,39]. Similarly, the pro-inflammatory cytokine IL1B can amplify inflammatory cascades through the NF-κB pathway, accelerating virus-induced lesion development [40–43]. PTGS2 encodes cyclooxygenase-2, which amplifies inflammatory responses through lipid mediator synthesis and is induced by inflammatory stimuli in various cell types, thereby potentially contributing to HPV-associated pathogenesis [44]. NFKB1, encoding the p50 subunit of NF-κB, is a critical regulator of inflammation and immunity in HPV infection, and its downregulation can be exploited by the virus to evade host immune responses, fostering viral persistence [45,46]. AKT1, EGFR, and CASP3 are crucial for regulating cell survival and proliferation. The serine/threonine kinase AKT1 is a key component of the PI3K pathway and regulates multiple cellular processes, including proliferation, migration, and invasion [47,48]. It is frequently upregulated in HPV-driven cancers, where it promotes oncogenic progression [49–51]. The E5 protein of HPV can directly bind to and activate EGFR, a tyrosine kinase receptor that promotes cell proliferation and differentiation, highlighting a direct viral mechanism to manipulate host cell machinery [52,53]. Conversely, CASP3, an executive molecule in apoptosis, is often downregulated as HPV lesions progress, allowing infected and transformed cells to evade programmed cell death [54–56]. TLR4, PPARG, JUN modulate the cellular environment. TLR4, a mediator of inflammation and tumorigenesis, can promote tumor growth and shape an immunosuppressive microenvironment post-HPV infection [57–59]. PPARG, a nuclear receptor involved in metabolism and immune regulation, can have its activity disrupted by HPV, leading to metabolic alterations like lipid accumulation [60]. JUN, a component of the activator protein-1 transcription factor, participates in the processes of cervical cell transformation [61]. In summary, this multifaceted target network highlights the potential of gut microbial metabolites to disrupt key pathological processes in HPV infection through coordinated effects on inflammation, cell survival, and the immune microenvironment.

GO enrichment analysis revealed that the 43 key targets were primarily involved in microbial signal recognition and cellular stress responses, including responses to lipopolysaccharide and reactive oxygen species. These processes were closely associated with membrane rafts, the NF-κB p50/p65 complex, and the AP-1 transcription factor complex. Membrane rafts serve as platforms for lipopolysaccharide-induced Toll-like receptor 4 signaling, which activates NF-κB and AP-1 to drive inflammatory gene expression [62]. Previous studies have shown that the HPV 16 E5 oncoprotein upregulates COX-2 through these transcription factors, contributing to cervical epithelial transformation [63]. Concurrently, the enrichment of response to oxidative stress and cellular response to reactive oxygen species reflects the redox imbalance in HPV pathogenesis. HPV E6 and E7 oncoproteins disrupt antioxidant defenses, leading to reactive oxygen species accumulation, DNA damage, and genomic instability that facilitate viral integration [64]. The enriched molecular functions, including heme binding and NAD(P)H-dependent monooxygenase activity, implicate cytochrome P450 enzymes in modulating intracellular redox status [65]. Collectively, these findings suggest that gut microbial metabolites may influence HPV infection by regulating lipopolysaccharide-mediated inflammatory signaling, oxidative stress responses, and their associated transcriptional complexes. KEGG enrichment analysis revealed that gut microbiota and their metabolites primarily exert regulatory effects on HPV infection mechanisms through five signaling pathways: the TNF signaling pathway, Toll-like receptor signaling pathway, C-type lectin receptor signaling pathway, and IL-17 signaling pathway in diabetic complications. Activation of the TNF signaling pathway can induce the secretion of proinflammatory cytokines, creating an inflammatory microenvironment conducive to persistent HPV infection [66]. Inhibition of the Toll-like receptor signaling pathway impairs host antiviral immunity, facilitating immune evasion of HPV [67]. The IL-17 signaling pathway, closely linked to immunity and inflammation, can accelerate lesion progression by promoting cell proliferation [68,69]. These pathways play critical roles in immune regulation, inflammatory amplification, and malignant transformation during HPV infection.

Pharmacological similarity and toxicological analyses of 13 gut microbial metabolites identified succinate as the most promising candidate related to HPV infection progression. Molecular docking further revealed that succinate exhibits a favorable binding capacity to IL1B, suggesting a potential role in IL1B-mediated inflammatory cascades. However, this computational finding requires cautious interpretation. Succinate is a small endogenous metabolite whose well-established immunomodulatory effects are primarily mediated through its specific cell surface receptor, SUCNR1 [70,71]. Activation of SUCNR1 by succinate can trigger pertussis toxin-sensitive Gi/Gq pathways, a mechanism that may relate to the pertussis disease pathway identified in our KEGG enrichment analysis [70]. In the cervical microenvironment during HPV infection, SUCNR1 is activated in macrophages, leading to altered metabolism and succinate accumulation. The released extracellular succinate further promotes IL1B production, amplifying the inflammatory response [72]. Thus, while our docking analysis raises the possibility of a direct succinate-IL1B interaction, its functional relevance remains to be experimentally validated. Alternatively, succinate may exert its effects on IL1B primarily through the canonical SUCNR1-mediated pathway, which is consistent with its established role in immune regulation.

In addition to succinate, other short-chain fatty acids (SCFAs) such as butyrate, propionate, and acetate also emerged from our screening. SCFAs exert immunomodulatory effects through multiple mechanisms, including activation of G protein-coupled receptors (GPCRs) and inhibition of histone deacetylases [73]. In neutrophils, propionate, acetate, and butyrate are the main SCFAs produced by fermentation by bacteria [74]. Bacteria of the phylum Bacteroides are strong SCFA producers and could be beneficial during viral infections, since Bacteroidetes produce high levels of acetate and propionate, whereas Firmicutes produce more butyrate [75]. Propionate contributes to epithelial barrier integrity and regulates inflammatory responses [76]. Acetate acts via its cognate receptor FFAR2 to potentiate innate immunity. In neutrophils, acetate-FFAR2 signaling enhances their recruitment to inflamed sites, activates the inflammasome, and promotes IL-1B secretion [77]. Butyrate reinforces the barrier function, exerts anti-inflammatory effects, and inhibits tumor growth through cell cycle arrest and apoptosis [78,79]. However, recent evidence indicates that sodium butyrate may suppress humoral immunity by inhibiting follicular T helper cell differentiation [80]. These findings highlight the complexity of butyrate’s role in immunity, which may vary with concentration, cellular context, and the specific arm of the immune response being engaged [81]. Regarding safety profiles, our toxicological evaluation indicated that among all metabolites screened, succinate emerged as particularly significant due to its favorable drug-likeness and acceptable toxicological profile. Among the short-chain fatty acids, butyrate and propionate exhibit favorable safety, with skin sensitization being the only predicted adverse effect. Acetate, however, showed potential for both skin sensitization and drug-induced liver injury, warranting caution. The differential effects and safety profiles of these SCFAs suggest they may hold translational potential in HPV management. Future studies should explore whether modulating SCFA-producing gut microbiota could influence the cervical immune microenvironment through the gut-cervix axis.

From the perspective of the MMTS network, succinate production is linked to the metabolic activities of diverse gut microbiota. It is primarily derived from *Succinivibrio dextrinosolvens* and *Succinatimonas hippei* (Proteobacteria), as well as *Bacteroides thetaiotaomicron* and *Parabacteroides distasonis* (Bacteroidota). Members of the family Veillonellaceae and *Acidipropionibacterium acidipropionici* (Firmicutes) may also contribute to succinate homeostasis through indirect metabolic pathways [82]. Changes in the abundance of these microbiota may influence the host’s local immune microenvironment through the “gut-cervical axis”. On the one hand, succinate could reach cervical tissues via the bloodstream, and through targeting IL1B, activate signaling pathways, including the TNF signaling pathway, Toll-like receptor signaling pathway, C-type lectin receptor signaling pathway, and IL-17 signaling pathway. This provides a theoretical basis for hypothesizing that succinate, once reaching cervical tissues via the bloodstream, could influence local immune responses [83]. However, how succinate is transported from the gut to the cervix, its local concentration, and its direct immunomodulatory effects in the cervical microenvironment remain unclear. Thus, while our findings support a potential “gut-cervix axis” in HPV infection, this concept requires direct validation, particularly regarding systemic metabolite trafficking and cervical immune regulation. On the other hand, dysbiotic mediators released by imbalanced gut microbiota can induce hormonal imbalance, leading to abnormal estrogen metabolism and disrupting the vaginal microecology, which could indirectly affect the outcome of HPV infection [19,84]. Taken together, our findings position succinate as a potential metabolic marker in HPV progression. Further research is warranted to elucidate the functional role of succinate in HPV pathogenesis and to determine whether modulating its levels could represent a viable strategy for intervention.

Higher dietary fiber intake has been associated with a reduced risk of HPV infection, and certain probiotics have shown promise in clearing HPV-related lesions [85]. These benefits may be mediated through prebiotic dietary factors such as inulin and fructooligosaccharides, which serve as key substrates for gut bacteria and are fermented into SCFAs, including acetate, propionate, and butyrate [86]. Building on this, targeting succinate-producing bacteria identified in our MMTS network, namely Proteobacteria, Bacteroidota, and Firmicutes, through probiotics or dietary modification may help reduce succinate levels at the source [87]. The bacterial strains identified as producers of succinate and other bioactive compounds in our MMTS network may themselves hold potential as next-generation probiotics. In addition, disrupting the interaction between succinate and IL1B could be worth considering. Developing small-molecule inhibitors that specifically block this interaction may inhibit IL1B-mediated inflammatory pathways. Currently, IL1B targeted therapies such as anakinra, an IL1 receptor antagonist, have been approved for inflammatory diseases [88], and their potential application in HPV-related persistent inflammation warrants further investigation. Another angle is SUCNR1, the receptor through which succinate signals [70,72]. Blocking SUCNR1 activation might prevent some of the immune dysfunction seen in the cervical microenvironment, like overactive macrophages and excessive cytokine release. Since SUCNR1 antagonists have shown potential in metabolic disease, it’s not a stretch to think they might be repurposed for HPV [89]. However, any of these strategies would need to be weighed carefully against safety concerns.

## 5. Conclusion

This study systematically mapped gut microbial metabolites relevant to HPV infection, identifying 10 core inflammatory and immune regulatory targets (IL6, AKT1, IL1B, CASP3, NFKB1, EGFR, PPARG, JUN, PTGS2, and TLR4) and their associated pathways, including TNF, Toll-like receptor, C-type lectin receptor, and IL-17 signaling pathways. Succinate stood out among the 13 screened metabolites for its combination of a favorable safety profile and predicted binding to IL1B (−5.66 kcal/mol). The identification of succinate-producing bacteria (Proteobacteria, Bacteroidota, Firmicutes) further links gut microbial activity to the proposed gut-cervix axis. These findings provide a testable framework for investigating how specific gut-derived metabolites might influence cervical immunity, and offer prioritized targets and pathways for future experimental validation.

## 6. Limitations

Despite the progress made in this study, several limitations must be acknowledged. First, the host targets were retrieved from public databases using the general keyword “HPV infection”, which may introduce inherent biases such as incomplete annotation and publication bias. Moreover, the obtained data were not stratified by viral genotype, patient gender, or age groups. Although our study identified universal core targets, their specific relevance to high-risk types such as HPV16 and HPV18 requires further investigation. Second, while we assessed drug-likeness, toxicological profiles, and performed molecular docking simulations to predict metabolite-target interactions, these findings are based on computational analyses. It is important to note that the associations identified between gut microbial metabolites and HPV infection targets are correlational rather than causal, and their directionality requires experimental validation to confirm the biological relevance and therapeutic potential of these interactions. Future studies utilizing stratified clinical cohorts and experimental approaches will be essential to substantiate these findings.

## Supporting information

S1 Data S1 Fig. Molecular docking assays for the core target IL1B and the positive control quercetin. S1 Table. Gene Ontology (GO) enrichment analysis of key target genes. S2 Table. KEGG pathway enrichment analysis of key target genes. S3 Table. Toxicological properties of the metabolites from the gut microbiota. All data were predicted using the ADMETlab 3.0 platform. For the classification endpoints, the prediction probability values are transformed into six symbols: 0–0.1 (---), 0.1–0.3 (--), 0.3–0.5 (-), 0.5–0.7 (+), 0.7–0.9 (++), and 0.9–1.0 (+++). S1 File. Abbreviations.
(ZIP)
