# Supplementary material for: The identification of metabolites from gut microbiota in HPV infection via network pharmacology
Source: PLoS One. 2026 Apr 10;21(4):e0346716. doi: 10.1371/journal.pone.0346716 (PMC13068282; doi:10.1371/journal.pone.0346716)
Supplement: S1 Data — S1 Table. Gene Ontology (GO) enrichment analysis of key target genes. S2 Table. KEGG pathway enrichment analysis of key target genes. S3 Table. Toxicological properties of the metabolites from the gut microbiota. All data were predicted using the ADMETlab 3.0 platform. For the classification endpoints, the prediction probability values are transformed into six symbols: 0–0.1 (---), 0.1–0.3 (--), 0.3–0.5 (-), 0.5–0.7 (+), 0.7–0.9 (++), and 0.9–1.0 (+++). S1 File. Abbreviations. (ZIP) [file pone.0346716.s001.zip › Supporting Information/S1 File.docx]

# Abbreviations

| **Abbreviation** | **Full Form** |
| --- | --- |
| ADME | Absorption, Distribution, Metabolism, Excretion |
| AKT1 | AKT Serine/Threonine Kinase 1 |
| BP | Biological Process |
| CASP3 | Caspase 3 |
| CC | Cellular Component |
| COX-2 | Cyclooxygenase-2 |
| DILI | Drug-Induced Liver Injury |
| EGFR | Epidermal Growth Factor Receptor |
| FDR | False Discovery Rate |
| FFAR2 | Free Fatty Acid Receptor 2 |
| GO | Gene Ontology |
| GPCR | G Protein-Coupled Receptor |
| HBA | Hydrogen Bond Acceptors |
| HBD | Hydrogen Bond Donors |
| hERG | human Ether-à-go-go-Related Gene |
| H-HT | Human Hepatotoxicity |
| HPV | Human Papillomavirus |
| IL1B | Interleukin-1 beta |
| IL6 | Interleukin 6 |
| IL-17 | Interleukin-17 |
| JUN | Jun Proto-Oncogene |
| KEGG | Kyoto Encyclopedia of Genes and Genomes |
| MF | Molecular Function |
| MMTS | Microbiota-Metabolite-Target-Signaling Pathway |
| MW | Molecular Weight |
| NFKB1 | Nuclear Factor Kappa B Subunit 1 |
| NF-κB | Nuclear Factor kappa-light-chain-enhancer of activated B cells |
| OMIM | Online Mendelian Inheritance in Man |
| PDB | Protein Data Bank |
| PI3K | Phosphatidylinositol 3-Kinase |
| PPAR | Peroxisome Proliferator-Activated Receptor |
| PPARG | Peroxisome Proliferator Activated Receptor Gamma |
| PPI | Protein-Protein Interaction |
| PTGS2 | Prostaglandin-Endoperoxide Synthase 2 |
| RMSD | Root-Mean-Square Deviation |
| SCFA | Short-Chain Fatty Acids |
| SEA | Similarity Ensemble Approach |
| SMILES | Simplified Molecular Input Line Entry System |
| STAT3 | Signal Transducer and Activator of Transcription 3 |
| STP | SwissTargetPrediction |
| SUCNR1 | Succinate Receptor 1 |
| TLR4 | Toll Like Receptor 4 |
| TNF | Tumor Necrosis Factor |
| TPSA | Topological Polar Surface Area |
| TTD | Therapeutic Target Database |
